# Tutorial. A Behavioral Analysis of Rationality, Nudging, and Boosting: Implications for Policymaking

**DOI:** 10.1007/s40614-021-00324-9

**Published:** 2022-01-26

**Authors:** Marco Tagliabue

**Affiliations:** Department of Behavioural Sciences, OsloMet - Oslo Metropolitan University, PO Box 4, St. Olavs Plass, 0130 Oslo, Norway

**Keywords:** Behavioral insights, Boosting, Freedom, Nudging, Policy, Rationality

## Abstract

As recent trends in policymaking call for increased contributions from behavioral science, nudging and boosting represent two effective and relatively economic approaches for influencing choice behavior. They utilize concepts from behavioral economics to affect agents’ concurrent suboptimal choices: in principle, without applying coercion. However, most choice situations involve some coercive elements. This study features a functional analysis of rationality, nudging, and boosting applied to public policy. The relationship between behavior and environmental variables is termed a “behavioral contingency,” and the analysis can include social and cultural phenomena by applying a selectionist perspective. Principles of behavioral control, whether tight or loose, may be exerted by policymakers or regulators who subscribe to paternalistic principles and may be met with demands of libertarianism among their recipients. This warrants discussion of the legitimacy and likelihood of behavioral control and influence on choices. Cases and examples are provided for extending the unit of analysis of choice behavior to achieve outcomes regulated by policies at the individual and group levels, including health, climate, and education. Further research and intervention comprise the study of macrocontingencies and metacontingencies. Advancing the understanding and application of behavioral science to policymaking may, therefore, benefit from moving from the relatively independent contributions of behavioral economics and behavior analysis to an inclusive selectionist approach for addressing choice behavior and cultural practices.

Governments are increasingly resorting to behavioral science for addressing their policy objectives (Benartzi et al., 2017). Behavioral science comprises an umbrella of disciplines that share a strong focus on behavior. Each of them contributes with their theories and approaches and this work aims to discuss the contributions of behavioral economics (BE) and behavior analysis (BA) to influencing behavior and policy using two cost-effective approaches that prompt better choices and decision making, nudging and boosting, which have been progressively adopted in policymaking (see Organisation of Economic Cooperation & Development [OECD], 2017a, b; Ruggeri et al., 2019). Like BA, BE aims at understanding human behavior with sufficient descriptive reliability by including empirical observations (i.e., data on how people behave) in research on choice and decision making. The basis of observation is behavior, and not attitudes, opinions, or intentions of the agents whose choices the researcher or policymaker want to understand and influence, especially in as much as these choices can be characterized by objective and predictable irrationality (Ariely, 2008) or yield suboptimal outcomes compared to normative theories of rational choice. For example, Thaler (2015) documented the origins of BE and his specific contributions to irrational phenomena such as choice anomalies, mental accounting (i.e., assigning different values when managing the same amount of money), financial decision making, and problems of self-control. Several studies have documented systematic and consistent deviations from optimal economic behavior in psychology (e.g., Hewig et al., 2011), BA (Herrnstein, 1990), and policymaking (Evans, 2017). Choosing is economic behavior because it represents the allocation of time and effort, both of which are scarce resources. Theories of rational choice do not account for observed deviations from optimizing outcomes. Satisficing describes these deviations better, as suggested by Herbert Simon (1956).

The ambition of the present study is twofold. First, to analyze the concepts of rationality, nudging, and boosting, as behavioral processes defined in BA terms, albeit starting from their formulation in BE. Second, see how they may be embedded in policymaking. By defining them in behavioral analytic terms it is possible to trace the real contingencies for maintaining or changing the behaviors of interest. Nudging and boosting can serve as corrective measures for irrational choices by affecting the arrangement of contingencies of behavior, which may raise concerns about the extent to which nudge policies retain freedom of choice (see Schmidt & Engelen, 2020).

The focus is on tactics and strategies to influence the environmental and social context of rational choice behavior, rather than on the inconsistency between cognition and behavior that characterizes the agent’s economic behavior and its pitfalls. Whitehead et al. (2014) counted 135 countries that have implemented behavior change policies in different areas, ranging from HIV prevention to healthy pregnancy initiatives, and from fertilizer use to police force reform; of these, 51 countries have applied centrally orchestrated forms of nudge-type policies applied to energy, charity, and public health, and other domains (see also Cesareo, 2018). Since then, the list of countries applying behavioral science to policymaking has most likely increased for at least two reasons: first, the establishment of “nudge units” around the world both inside (e.g., the Behavioural Insights Team in the United Kingdom, the Office of Information and Regulatory Affairs under former President Obama’s administration) and outside (e.g., Ideas 42, the World Bank) government; second, the convincing results that their initiatives had on policy (see Organisation for Economic Cooperation and Development (OECD), 2017a, b), which have been further boosted since March 11, 2020, when the World Health Organization declared COVID-19 a pandemic and policymakers around the world implemented behavioral principles for containing and preventing further spread of the virus (e.g., Michie et al., 2020).

This study is structured in the following way: rational choice is first defined according to the BE approach and then analyzed including relevant terms from behavioral science at large. The concept of *rationality* is important when interpreting models of decision making and choice behavior, and its assumption comprises the discriminating factor between neoclassical economics and BE (see Binmore, 2008, for a thorough account of the topic).

Next, a selectionist approach grounded in behavior analytic principles emphasizes the role of the environment in supposedly irrational choices. In BA terms, the selected behaviors may be functionally related to either rule-governed behavior, shaped by the present contingencies, or both. Nudging and boosting rely on changing environmental contingencies or introducing socially valid rules, rather than being rooted in a selectionist perspective where the importance lies on the consequences of the behaviors of interest. Nevertheless, boosting may program different effects on the contingencies that precede and follow choice depending on whether the approach is informed by BE or BA.

Finally, some ethical considerations of behavioral influence and control are discussed, before concluding with an outline of practical implications for behavioral designers and policymakers. These implications affect macrocontingencies and metacontingencies, which identify the relations of arranging the environmental and social contingencies of behavior in larger groups and cultures, respectively. According to Taleb (2018), “What is rational is that which allows for *survival.* . . . Anything that hinders one’s survival at an individual, collective, tribal, or general level is, to me, *irrational*” (p. 220; emphasis in original). Yet, what is rational for one agent may not be rational for another, or for the group to which they belong.

## A Behavioral Economics Approach to Rational Choice

The concept of *economic behavior* corresponds to the behavioral repertoire that is composed of (1) the allocation of scarce resources (i.e., economics, in its broadest sense; Nicholson, 1992), which include money time, labor, behavior, and (2) the environmental and social context in which allocation (choice) is situated. Economic behavior is regarded as a subset of institutional economics concerned with the interlock of several agents’ choices (see Commons, 1934/1989). This study embraces a cultural-selectionist perspective rather than limiting the analysis of economic behavior to the individual or ontogenetic level. As Katona (1953) used the term, “economic behavior” identifies behavior that concerns economic matters and does not refer to the behavior of the “economic man” (p. 309); hereafter it will be considered a form of choice given the economic matters (and choices) of others.

From an operational standpoint, economic behavior is the product of generalized choice behavior, which intends the recurrent allocation of scarce resources by individuals and groups across situations. Cognitive theories of decision making, judgment, and problem solving are alternative explanatory accounts derived from the cognitive underpinnings of operant or rule-governed choice behavior (e.g., Crozier et al., 1997; Sweller, 1988). Although BA scholars may endorse experimental procedures and findings from BE, they are also wary of flawed theories resting on circular reasoning and the causal status of psychological constructs, thus losing their explanatory power. Eminent theories within the BE approach have been scrutinized concerning definitions, procedures, and experimental results. For example, the critique of Staddon (2018) of prospect theory, which is a theory of decision making developed by Kahneman and Tversky (1979), addressed the issues that several reframed concepts lack explanatory power, experimental findings in one third of the participants contradicted the theory, and he presented general arguments for the coexistence of functional and mechanistic models for scientific understanding (Amd, 2018).

*A Behavioral Model of Rational Choice* by Simon (1955) outlines how choices tend to be merely satisfactory rather than optimal. This statement is consistent with a selectionist perspective. *Homo economicus* represents utility optimization as the highest possible subjective value for the agent, whose ultimate metaphysical representation is the Olympian model described by Simon (1983). The *demon* of Laplace (1814/1951) preceded Simon’s *Homo economicus* by some years:

We may regard the present state of the universe as the effect of its past and the cause of its future. An intellect which at a certain moment would know all forces that set nature in motion, and all positions of all items of which nature is composed, if this intellect were also vast enough to submit these data to analysis, it would embrace in a single formula the movements of the greatest bodies of the universe and those of the tiniest atom; for such an intellect nothing would be uncertain and the future just like the past would be present before its eyes. (p. 4)

Laplace never called it a demon, but with this archetype of a superhuman powered by infinite computing capacities, economic behavior is always rational and bears the most beneficial consequences for the agent. Laplace’s demon is impossible, given the principle of computational irreducibility. A computer that could follow and predict everything in the universe would need to be as big as that universe; thus, models that approximate are the best that can be achieved.

The notion of *utility* in economics has changed since its first formulation in terms of hedonism by moral philosophers Jeremy Bentham (1748–1832) and John Stuart Mill (1806–1873). However, it remains mostly an interdependent measure. If an individual or a group act to gain more utility for themselves or their group, this may directly or indirectly imply that contingencies may be altered in a way that lowers the utility of another individual or group through the former’s cumulative behavior and its economic consequences. For example, if going to the park is preferred to going to the mall on a sunny weekend, and this difference in utility is shared among the larger community, it follows that going to the park may not entail the same pleasure for everyone. If the community has access to only one park, there might not be enough personal space for everyone to make it either worthwhile or an optimal choice, which in the present example represents a common-pool resource. According to theories of rational choice, however, rational agents can predict the crowds in the park and calculate their loss of comfort: they avoid the park and optimize their utility, regardless of the other agents’ utilities. The interrelation between agent’s and others’ choices is typically omitted in models of rational choice that are concerned with the individual unit of analysis. Nevertheless, the behavior of utility optimizers is affected by selfish concerns, not by the utility of other agents. This discrepancy represents a social dilemma (e.g., see Poundstone, 1992), wherein individual and collective utility functions diverge.

Ostrom and her colleagues (e.g., Ostrom, 1990) studied groups governing common-pool resources and observed with ethnographic methods how rational choice is a form of behavior that does not necessarily need to be in line with the zero contribution thesis. According to this thesis, “individuals cannot overcome collective action problems and need to have externally enforced rules to achieve their own long-term self-interest” (Ostrom, 2000, p. 137). Thus, the utility of individuals is ordered higher than the utility of the group to which they belong, unless the group is sufficiently small or the coercion is imposed (see also Olson, 1965). Rational choice may “evolve” by being consistently selected, even though variation may occur as other group members’ strategies change. Groups managing common-pool resources are successful if the positive outcomes are shared and maintained beyond the group’s boundaries (i.e., if they feature high between-group cooperation), whose relations should be appropriately regulated by eight core design principles (Ostrom, 1990; see also Wilson et al., 2013), and not exploited internally (i.e., if they feature high within-group competition; see Wilson, 2015). The work by Ostrom has been criticized for failing to consider more complex systems than the small-scale commons managing natural resources used for developing the eight core design principles (Stern, 2011). Block and Jankovic (2016) claimed that Ostrom’s work rested on false premises because she considered governmental protection a condition for private property when distinguishing it from the commons. Moreover, the core design principles may feature limitations in applicability to other groups (e.g., who do not manage common-pool resources) in real-world applications. More recently, this line of work has informed processes of co-design for improving health care, which was retrospectively related to system understanding and mapping, upholding democracy, and regulating participation (Robert et al., 2021).

Choice is the main dependent variable in BE studies. Camerer et al. (2005) described choice as a process people use to select an action. Choice is usually analyzed on a single-subject scale and features either known-and-certain or risky-and-uncertain consequences for the agent (Angner, 2016). This allocation is typically made between two or more alternatives. However, Martin et al. (2006) noted that choice may apply to a subset within a full set of alternatives, one single item in the menu (any item or the preferred one), a cognitive process involving mental computation (reality doubling), or the more abstract ability to choose. The last item is also reality doubling, for the only basis for concluding about ability is the actual behavior exhibited. The term “menu” represents this concept and stands for the whole array from which an agent may choose. It features two properties that characterize most choice situations: (1) mutual exclusivity, when choosing one alternative involves discarding the other(s), and (2) exhaustiveness, when choosing not to choose is not an option (Angner, 2016). Although normative models postulate that more alternatives are consistently preferred to fewer, the problems of *overchoice* or choice overload (e.g., see Schwartz, 2004; Toffler, 1970) are examples of how theories of rational choice may be contradicted (e.g., the adage “less is more”). Agents being exposed to the overwhelming effects of choice experience a threat to their utility optimization efforts. Policymakers are often aware that introducing more choices increases both the likelihood that at least one of them meets the agents’ preferences and the burden that they entail. Hence, Johnson et al. (2012) suggested simplifying the choice attributes or the number of options. For example, harnessing the power of defaults options, which are preselected for the agent and need to be actively acted upon to change them, has been demonstrated in domains ranging from investment (Cronqvist & Thaler, 2004) to organ donation (Johnson & Goldstein, 2003).

Another classic example according to Mullainathan and Shafir (2013) is eating everything there is, instead of following a healthy diet. If one grew up in poverty, he or she knows enough to get it while available; if one’s childhood and youth were relatively affluent, this makes no sense. Although rationality lies in the eye of the beholder, this covers only half the point that must be made. Whether a choice is rational or not is a normative question that is not pertinent to the process of choosing per se. To Simon, decisions “bridge the distance between rationality and behavior” (see Barros, 2010), which is another case of inferring mental process components of choosing when all available data are behavioral.

Nevertheless, the term “rational” may be preceded by an adjective, to specify the contextual appropriateness of best choices. Choice behavior can be characterized as *objectively* rational, *deliberately* rational, *organizationally* rational, and *personally* rational (Simon, 1957). In general, survival contingencies for an individual or group that may determine their demise are not rational. Thus, a descriptor such as *rational* poses a ubiquitous threat of slipping into normativity, whose justification is unsupported unless properly contextualized, and may lead to the conclusion that the concept of rationality is mostly superfluous. In fact, BA takes a different stance based on descriptions of natural and physical processes whose rational value is a function of the consequences of choice behavior.

## A Behavior Analytic Account of Rational Choice

Choice is the operant behavior of doing one thing rather than another when two or more options exist. In BA, choice is understood as the distribution of operant behavior among reinforcing alternatives (Pierce & Cheney, 2008), and choice behavior implicitly concerns how individuals allocate their time to response options (Fisher & Mazur, 1997). Similar to other instances of response allocation, choice may be affected by antecedent and consequent events. Moreover, the potential reinforcing value of present and future commodities is affected by time constraints. For example, the process of discounting occurs when perceived value is greater in the present than in the future. For example, policy and regulation of tobacco-based products are particularly prone to incorporating this process, as a systematic review with network analysis has shown that smokers discount future events more steeply and, in turn, higher discounting predicted future smoking (Barlow et al., 2017).

Choice is dependent on its encompassing environment and represents a function of the physical and social contingencies that program for acting on the environment. For example, choice behavior may be under concurrent control of contingencies and schedules of reinforcement, and the agent’s verbal rules (Fisher & Mazur, 1997). Moreover, any instance of choice behavior can be seen as a product of a cultural lineage, transmitted and selected within a group. The field of BA has a long-standing tradition of studying choice behavior with animals (e.g., McSweeney & Murphy, 2014). Laboratory studies with pigeons have demonstrated that free choice is consistently preferred to forced-choice behavior (e.g., Catania & Sagvolden, 1980; Cerutti & Catania, 1997), as long as the magnitude of reinforcement is equal to or greater than in a corresponding forced-choice situation (see also Rost et al., 2014). This may hold true in empirical studies of human subjects, as theories of rational choice suggest. In turn, public policies inspired by the ideals of libertarianism entail increased behavioral variability among their receivers, including erring and affecting their learning histories.

Choice can be affected by selecting contingencies both at the individual level (i.e., ontogenetic [Skinner, 1981]) and at the group level [i.e., cultural; see also Couto, 2019]). In the latter, behavior is maintained and transmitted as old group members are replaced by new ones (e.g., across generations; Sandaker, 2009). From a selectionist perspective, cultures evolve by arranging contingencies in the social system that can override the control exerted by competing contingencies of selection at the individual level (Sandaker, 2009; Skinner, 1981). Social and nonsocial contingencies represent important units of selection to understand behavioral patterns in community settings (Abreu Vasconcelos, 2013), yet their effects can be observed at different levels. Thus, cultural practices that ensure access to commodities are selected if they positively reinforce the behavior of the individuals and the group. The selecting contingencies for cultural practices are both material and socio environmental events. Whereas this level of analysis and intervention comprising a culture-behavior science of societal challenges has been investigated through several conceptual studies and some basic laboratory research, behavior analysts seem not to have yet succeeded in exporting it outside the lab (see Mattaini, 2019).

Another selecting contingency is given by the generalized conditioned reinforcers: money represents the example to which we may most easily relate (Skinner, 1953). Because choice behavior is a form of operant behavior and a function of aspects of its environment (e.g., Skinner, 1938), it cannot be addressed in isolation from its sustaining context, which represents one topic where psychology is consistent with BA. The social context in which choice behavior and its consequences take place is a product of selection, and evolutionary processes may occur in the self-regulation of organisms, their behavior, and environmental events over time (see Glenn & Field, 1994). Differently from exponents of neoclassical economics, not only did Simon (1983) innovatively take the context into account, but he also placed choice behavior into an evolutionary perspective.

The matching law (Herrnstein, 1961) is another description of how context matters in choice behavior (Bickel et al., 1995). According to the matching law, the descriptive relation between relative choice rates and the rate of relative positive consequences available is hyperbolic and may be compared to the principle of price elasticity of demand in microeconomics. Nevertheless, the original matching law was eventually falsified by McDowell et al. (2017) in favor of an alternative (although not exclusive) evolutionary theory. The prediction concerning the descriptive adequacy of matching did not receive empirical support and models based on alternative concepts such as allocation, induction, and contingency (Baum & Davison, 2014) have been advanced for explaining deviations from the matching law. Moreover, in his article on rational choice theory, Herrnstein (1990) suggested considering the notion of utility in economics in a similar way to the concept of reinforcement in behavioral psychology. As neither utility nor reinforcement may be directly observed, their effects must be inferred from choice behavior. Although theories of rational choice are axiomatic and normative with respect to optimizing reinforcement, in several cases they fail to describe actual behavior. In turn, it is possible to improve current policies by improving the predictions about their effects: for example, inertia as a hinderer of retirement savings subsidies or income tax (Chetty, 2015).

Rational and irrational choices are dynamic processes of contextual decision making (Furrebøe & Sandaker, 2017) and are a function of perspective-taking. Decision making refers to “how current stimuli and learning history combine to determine choice” (Fantino, 1998, p. 355). Decisions are viewed as the result of the process of operant selection, which may constitute a case of reality doubling, according to the view in BA. The agent may act rationally given his or her learning history and perception of contextual variables, whereas an observer may consider the same behavior irrational given his or her learning history and perception of concurrent contextual variables. The concept of bias was proposed to account for recurrent patterns of maladaptive deviations from optimal choice. However, biases have had an important evolutionary function, both for the phylogenetic and ontogenetic advancement of our species, in particular when dealing with symbolic behavior (see Tagliabue et al., 2021).

A behavioral definition of choice entails “the presence of two or more salient discriminative stimuli [S^D^], at least one of which is a relatively effective S^D^, as compared to others in that situation” (Martin et al., 2006, p. 4). Thus, choice is subject to selection by consequences. Although the normative value of rational choices may be debated, “irrational” self-destructive choices may attract more objective agreement on their underlying contingencies. Substance abuse and addiction represent examples of temporal and probability discounting of negative outcomes, and are frequently interpreted as lack of self-control (Rachlin, 2000): a special form of choice that entails cooperation with “one’s past self” (i.e., in relation to one’s previous choices). For example, smoking cigarettes can be seen both as the smoker’s rational discretion to attain his or her own welfare and irrational behavior that threatens social welfare for the public health costs that it entails (see Solek, 2014). Policymakers are particularly attentive to the latter, although it may be limiting to exclude the former from a functional analysis of the reinforcing effects of smoking.

Rachlin (2016) emphasized further that the difference between self-control and social cooperation is “one of degree and not of kind” (p. 249), wherein the latter requires two or more agents whose choices are interdependent. However, both self-control and social cooperation comprise extended patterns of choice that may evolve by pattern selection: a form of learning that resembles multilevel selection in biological evolution (Rachlin, 2016). The primrose path (Rachlin, 2000, 2002) represents an example of choice behavior that contradicts the axioms of Benthamite utility optimization (i.e., tending towards the increase of the agent’s overall happiness). Like the smaller–sooner/larger–later paradigm, it introduces immediacy and delay into the equation with other values than in a utility optimization equation. The fatal consequences of such choices, which manifest themselves at any given time in the future, are still foreseeable when the (bad) choice behavior occurs. Similar to how Shakespeare coined the term in 1609 in *The Tragedy of Hamlet, Prince of Denmark* (Act I, Scene 3), Herrnstein and Prelec (1992) used the primrose path for explaining “How can you always choose the best among all available alternatives and still end up in a worse state than when you started?” (Rachlin, 2000, p. 72).

Complex ambivalence refers to the temptation to choose the most gratifying option in the short run, although it bears the cost of hindering a better future outcome. Contrary to simple ambivalence, it may not suffice to commit to a selected strategy because choice is not restricted to clearly defined alternatives and includes attaining a vaguely defined abstract state (Rachlin, 2000). Whereas complex ambivalence was originally formulated to exclusively address addiction behavior to alcohol, drugs, and gambling, it may apply to other choice behaviors characterized by complexity. Examples can be drawn from policies targeting financial literacy (e.g., paying all debt or just the minimum on one’s credit card) and conservation of the environment: juggling the short and long-term cost-benefit dilemma embedded in climate change initiatives. These and other instances of complex choice behavior represent instances in which nudging and boosting may represent effective techniques for informing policymaking and correcting the course of action.

### From Macrocontingencies to Metacontingencies in Policymaking

The term “macrocontingency” was first introduced in 1978 as a unit of analysis of sociocultural phenomena but lacked a functional definition (Ulman, 1998). Macrocontingency was defined by Ulman (2006) as “*the conjoint actions of two or more individuals under common contingency control*” (p. 95; emphasis in original)*.* However, Glenn (2004) used the term in a different way than Ulman did (Glenn et al., 2016; Mattaini, 2006; Ulman, 2006). The concept of macrocontingency in Ulman (2006) is invoked “to indicate when there is no direct contingency between the operant emitted by any individual of the group and the observed cumulative social effect” (Abreu Vasconcelos, 2013) (p. 33). Thus, the term “macrobehavior” is preferable and more precise for the relationship is indirect; it refers to “[s]ocially-learned operant behavior observed in the repertoires of several/many members of a cultural system” (Glenn et al., 2016, p. 18).

Interventions based on behavioral science may contribute in an impactful and timely fashion toward increasing environment-conscious choice behavior, among other possible applications. The data may lay the foundations for developing better-informed and evidence-based policies for larger groups and communities, improving sustainability for both behavioral- and policy-level interventions. It is possible to leave the aspect of interaction (represented by the interlocking behavioral contingencies; please see details below) out of the analysis: this choice of method is certainly more economic, but less desirable, for it may limit users’ involvement because of their interdependence.

Macrobehavior is a functional differentiation from the concept of *metabehavior*, which identifies the behavioral class of cultural practices (Glenn, 2004; Mawhinney, 1995). From a structural standpoint, macrobehavior represents the sum of individual behavior, whereas metabehavior is something different (or greater) from the result of the interaction between agents. The behaviors of several agents in the macrocontingency are usually disconnected from one another, and no lineage may be formed (Houmanfar & Rodrigues, 2006). Cultural-behavioral lineages are recurrent behavior, emerging from replicating socially acquired behavior within and beyond (in terms of survival) the social group (Glenn et al., 2016).

A *metacontingency* is a conceptual tool (Todorov, 2006) that comprises a contingency of cultural selection (Glenn, 2004). It made its first appearance in an account by Glenn (1986) of social contingencies described in *Walden Two* (Skinner, 1976) and it describes a functional relationship between a culturant and a selector (Glenn et al., 2016). The culturant is functionally equivalent to the operant (Reese, 1966), but the unit of analysis is social behavior, instead of individual behavior. The culturant is composed of interlocking behavioral contingencies and their resulting aggregate product, whose production is dependent on the former. The selector can take the denomination of a receiving system (whenever applied to organizational settings; cf. Houmanfar & Rodrigues, 2006) or a selecting environment, which can be social. The selector is responsible for the survival and transmission of the culturant. This feedback loop often requires higher-ordered decision-makers or the members of the group themselves to track outcomes and select different rules that may affect the interlocking practices within the organization. For example, Biglan interpreted the economic concept of negative externalities (i.e., an indirect accompanying negative consequence that is not economically accounted for) as metacontingencies for influencing research and policy on chemical pollution (see also Newland & Bailey, 2017, for behavioral and policy considerations of methylmercury).

Questions have been raised concerning the practical relevance of the concept of metacontingencies (e.g., Mattaini, 2004, 2006), and whether a new unit of analysis in addition to the behavioral contingency is needed for interpreting social phenomena. In a review of 30 years of publications on metacontingencies, Zilio (2019) concluded that there was no applied work in which a metacontingency was necessary. One limit of applicability was partially addressed by analyzing metacontingencies in behavior change experiments that bore social implications (Tagliabue & Sandaker, 2019). Although a metacontingency may not be strictly necessary, the cumulative effects of the intervention may be interpreted in terms of macrocontingencies. It seems legitimate to question whether metacontingencies are needed at all to analyze cultural phenomena, or whether the analysis of rule-governed behavior of groups and the cumulative effects of behavioral contingencies may suffice (Krispin, 2016; Sandaker, 2004; see also Aguiar et al., 2019). For example, Baum (2000) presenteds an analysis that systematically avoids concepts beyond basic behavioral principles. Verbal behavior and, in particular, rule-governed behavior are responsible for influencing social contingencies. These are maintained by the members of a group who work with one another, and the cultural practice resulting from their interaction cannot be reduced to the mere sum of their individual behaviors (Zilio, 2019).

## Nudging and Boosting: Interventions of Choice Architecture

Nudging refers to a form of contingency management (da Rocha & Hunziker, 2020) for altering the environmental and social antecedents of choice behavior, without changing how punishments and rewards are distributed. Nudging has a short history, but a long past: although it was formalized and popularized as a technique of behavioral influence only in 2008 (Thaler & Sunstein, 2008),^1^ Rachlin and Green (1972) “nudged” rational choices among their experimental subjects (i.e., pigeons) some 35 years earlier, helping them commit to attaining larger rewards. Inspired by and consistent with the “heuristics and biases” research program (Kahneman, 2003; Kahneman et al., 1982; Tversky & Kahneman, 1974), nudging helps policy- and decision-makers to overcome possible cognitive shortcomings and consequence misjudgments by providing readily implementable courses of action. Examples of nudging include introducing graphical or verbal warnings, eliciting implementation intentions, sending reminders, selecting defaults, leveraging social norms, and developing precommitment strategies (Sunstein, 2014a). As an example of the last item in this non-exhaustive list, unsafe activities and the resulting risk of being exposed to violent crime decreased by 50% among youths in Cape Town by introducing a “Safety tool” app for planning recreational social activities (e.g., attending a football match; Ideas42, 2014).

Nudges assume the existence of dual cognitive processing systems (Kahneman, 2011): they include Automatic System (System 1) nudges and Reflective System (System 2) nudges. System 1 nudges comprise “the set of interventions aimed at overcoming the unavoidable cognitive biases and decisional inadequacies of an individual by exploiting them” (Rebonato, 2012, p. 84). However, standing from a representative sample of U.S. respondents, System 2 nudges were perceived more positively and should be preferred (Sunstein, 2016b); these are educative nudges (comparable to boosts, which are introduced in the next section) and are “specifically designed to increase people’s capacity to exercise their own agency” (p. 7). From a behavior analytic perspective, System 2 nudges may transfer behavioral control from direct-acting contingencies to rules (Tagliabue et al., 2017). Nudging influences choice behavior that is inconsistent with the prescriptions of theories of rational choice, supposedly without exerting coercion (see the discussion section for a more thorough analysis) rather than resorting to cues for responding to relational frames (Tagliabue et al., 2019).

According to a behavior-analytic perspective, nudging identifies the disposition of behavioral contingencies, programming for the availability of reinforcement from the physical or social context. When including a social component, nudges are behavioral interventions that program positive consequences for both the individual and the group (Krispin, 2021). Once configured in a certain way, the environment influences choice behavior accordingly. A *neutral* choice architecture would be an oxymoron and is hardly possible; even a random placement affects choice (Thaler et al., 2014). Thus, the term “choice architecture” refers to the organization of the context in which people make decisions (Thaler et al., 2010).

The problem raised by having an agent as a choice architect reflects a parallel issue in nudging research concerning the duality verb-noun embedded in the English word *nudge*. If *nudge* is read as a noun, it resembles the role of an antecedent term in a three- or four-term contingency model. The emphasis is on the instrumental aspect: a nudge should affect *means*, rather than *ends*, and this makes its use more permissible, according to Sunstein (2014b). On the other hand, if *nudge* is intended as a verb, it may signify one of many “means of bringing behavior under the control of wide and abstract reinforcer contingencies” (Rachlin, 2015, p. 198). Although the aim of nudging is to modify the consequences of a target behavior, it does so by altering situational factors affecting choice behavior. The emphasis on softly manipulating ends, and not means, raises questions on the ethical legitimacy of nudges, even if they are intended for serving the greater good (Sunstein, 2014b). The greater good may be the agents’ interests, the community that draws a benefit, or society altogether by solving “wicked problems” such as pollution or poverty.

As nudging represents a clear example of physical or social contingency design, a more accurate description of its content is provided by its precursor term “libertarian paternalism,” which represents a philosophical position that captures the tension between self-determination and coercion. “Paternalism” is the idea that somebody (e.g., a policymaker concerned with achieving rational choices among the recipients of a policy) knows best what constitutes a good outcome for somebody else than they do themselves, and so should act to make this come about, even without their consent. A “libertarian” form of paternalism softens this position by removing coercion and allowing the receiver of the paternalistic act to change the course of action. The task of defining a nudge has attracted increasing interest from several disciplines, and many formulations are available: from an attempt at influencing judgment or choice by cognitive exploitation (Hansen, 2017) to subjectively paternalistic ways of rational persuasion (Hausman & Welch, 2010), and means of behavioral control of long-term contingencies (Rachlin, 2015). Nevertheless, nudges aim at altering behavioral responses by serving as environmental cues. For example, the fight against the COVID-19 pandemic features several nudging and other behavioral interventions for public health policy (e.g., Van Bavel et al., 2020), ranging from signposting hand sanitizers, reminding of wearing a face mask and washing hands, displaying visual cues for maintaining physical distance. With respect to informing public health policy and social marketing, nudging increased COVID-19 vaccinations in the form of text-based reminders improving the vaccination salience and easiness (Dai et al., 2021). However, there have been cases where nudging did not have the desired effects, for example concerning people’s intentions to comply with public health guidelines (Sanders et al., 2021) or the discounted effects of behavioral messages on compliance (Hume et al., 2021).

Nudges exploit contextual information to alter the likelihood of responding in line with the designer’s intentions or preferences. Similar to the menu, which represents a subset and a specific arrangement of the physical environment described by Skinner (e.g., 1953), choice architecture features the prearrangement of contingencies of the choice-sustaining environment (Simon & Tagliabue, 2018). This environment represents the “givens”: they lay the premises under which choice behavior takes place and can be affected in a paternalistic way (Simon, 1957). Without this paternalistic element, the concept of nudging is not relevant.

Cesareo (2018) maintains that the effects of nudging are limited to influencing the antecedents in the behavioral contingency: they may serve as discriminative stimuli, motivating operations, or both. A nudge that operates as a discriminative stimulus guides behavior (Rachlin, 2000), whereas motivating operations have two defining effects: they alter the effectiveness of reinforcers (or punishers) and the frequency of operant response classes related to those consequences (Laraway et al., 2003). Likewise, nudges may retain the same effects on choice behavior. However, they may hardly do so at the same time, for motivating operations alter the value of a possible behavioral consequence and affect behavior associated with these consequences. On the other hand, discriminative stimuli signal the availability of certain consequences because of a learning history with (frequently differential) reinforcement. Thus, agents can be nudged into engaging in choice behavior of which they have never experienced the consequences.

Because there is a mutual interaction between behavioral contingencies and an agent’s learning history, but also biological conditions and stimulus situations (Laraway et al., 2003), nudges may assume educative value. Although nudges may lead to learning as a byproduct of continuous exposure to similar contingencies (e.g., The U.S. Department of Agriculture’s MyPlate for healthy eating or the European Union’s Ecolabel certification for sustainable consumer choices), they were not introduced primarily as an educative tool. This aspect represents one of the main limitations of nudging and is an argument for developing an alternative to nudging that is methodologically more empowering for the agent, called boosting.

### Boosting: Educative Nudges and Reinforcement

The term “boosting” refers to two distinct approaches informed by BE (Grüne-Yanoff & Hertwig, 2016; Hertwig & Grüne-Yanoff, 2017), and BA (Crow, 2017): although resorting to the same term, they represent a subcategory and a complement to nudging, respectively, and entail different procedures. According to the former approach, which from this time forward is termed “BE boosting,” boosts are viewed as empowering means of behavior modification and control. They represent a critique of the self-serving purpose of nudges and coincide with “educative nudges” (Sunstein, 2016a). According to the latter approach, which is herein referred to as “BA boosting,” boosts strengthen the effects of nudges by programming the delivery of positive reinforcement.

BE boosting stemmed from a different research program than nudging: namely, Gigerenzer and peers’ work on the simple heuristics program (or fast-and-frugal heuristics, in Hertwig & Grüne-Yanoff, 2017). The simple heuristics program (e.g., Gigerenzer et al., 1999) represents an empirical approach to bounded rationality based on ecological rationality. It includes the conceptual commitments and their underlying assumptions that produce good (enough) choice behaviors in a given context (Grüne-Yanoff & Hertwig, 2016), and a framework for performance science (Raab & Gigerenzer, 2015). The simple heuristics research program was advanced to overcome the shortcomings of the effects of bounded or procedural rationality (e.g., Barros, 2010), formulated some decades earlier by Simon (1955). BE boosting extends the processing control of System 2, preparing for recurrent choice behavior under similar circumstances. BE boosting is particularly suitable in policymaking and has been resorted to increasing people’s risk literacy and decision skills in the uptake of cancer screening (Hertwig, 2017). Moreover, BE boosting extends the effect of nudges, specifically as they represent one-time fixes that may not work in different environmental contingencies.

In the eyes of the behavior analyst, BE boosting does not seem to substantially differ from establishing contingencies of operant behavior, as it entails learning through local environmental changes. Conversely, BA boosting aims at modifying choice behavior rather than its underlying cognitive process, and features the delivery of positive reinforcement to maintain it (Crow, 2017). The models of System 1 and System 2 represent the difference in scope between BE boosting and BA boosting. Whereas BE boosting raises attention on the need to target the underlying processing system to change a specific behavior, BA boosting focuses on the function and structure of choice behavior and neglects the process responsible for establishing a functional relation between behavior and environment. Hence, BA boosting aims at changing behavior through establishing different sets of contingencies. To work, BA boosting requires that the relevant behavior encounters the contingencies of reinforcement.

According to BA boosting, boosts refer to any positive consequences programmed in the contingency that sustain a modification of behavior, which is preceded by an antecedent in the form of a nudge. The simplest differentiation between nudging and boosting is that the first corresponds to prompting and the latter corresponds to the impact of reinforcement (Crow, 2017). Although behavior analysts may find the concept familiar, BA boosting possesses the merit of providing an operational account of the conditions for creating long-lasting behavior change once the effects of nudging fade out. For example, only 8% of interventions a megastudy (i.e., a massive field experiment testing out several concurrent behavioral interventions in the same population and in a comparable way) aimed at informing policy for encouraging physical exercise were still found measurable and significant after 4 weeks from rollout (Milkman et al., 2021).

Both BE and BA boosting are tools for behavior-based interventions. Although they are the result of different research traditions, there seems to be no reason why their effects should not be combined. Both forms of boosting represent not only a tactic for learning possible approximations to optimal choice behavior, but also for enhancing the long-term effects of nudging in the context of policymaking. This is one of the possible applications of behavioral insights.

## Discussion

### Behavioral Insights: Behavioral Science in Policymaking

Behavioral insights refer to the evidence-based application of concepts and methods of behavioral science to public policy “to provide better and more effective public policies” (OECD, 2019, p. 44). Behavioral insights represent an alternative approach to traditional policymaking, which assumes rational decision-making agents and does not consider the limits of our (flawed) choice behaviors (OECD, 2019). According to Ly et al. (2013), there are several tools that public policymakers can choose from to promote desired behavior change: regulation and restrictions, incentives (monetary and nonmonetary), information and education, and nudges and “nudge-type” strategies (in Cesareo, 2018). For example, Volpp et al. (2021) highlighted the discouraging effects of offering monetary incentives for COVID-19 vaccination, which may be associated with undesirability, unpleasantness, and unworthiness, and recommended resorting to contingent nonfinancial incentives instead. Nudge strategies have gained momentum in recent years and can be used both as a direct policy tool, wherein policymakers affect the contingencies first-hand, and indirectly, such as creating frameworks for nongovernmental organizations or other types of community services to implement nudges (Mont et al., 2014).

Policymaking may take the form of rule governance or verbal governance of behavior (Vargas, 1988) targeting adherence to social norms. Thus, nudges can influence the agent’s choice as a function of other agents’ choices. Examples of this class of nudges include providing (truthful) information about previous or current behavior in relevant reference groups, such as a neighbours’ electricity consumption, which increased energy efficiency (Costa & Kahn, 2013). Nevertheless, social norm nudges need not be limited to sharing information on what members of the agent’s reference group *do* but can also share information on what they *think* should be done. Thus, adhering to the rule stating that bills should be paid no later than on their due date may have the same effect as if the agent’s reference group subscribed to the same attitude, albeit without testing the behavior. These examples do not program for rational behavior per se but set the occasion for complying with the rule that describes the outcomes associated with either rational choice (i.e., environmental and economic savings for reducing one’s electricity consumption and avoiding a late-payment fee in addition to the amount of the bill). In another study by Hallsworth et al. (2016), social norms were exploited for reducing overprescriptions of antibiotics among family doctors in the UK, wherein the reference group was composed of other local family doctors. The results featured a 3.3% reduction of antibiotic prescriptions, contrasting the growth of antimicrobial resistance, and relieving public health of increased mortality, sickness, and cost of care.

However, the conditions for analyzing cultural phenomena may not always be met; for example, because there is no interaction or coordination among the agents. Thus, nudging in policymaking may be viewed as an intervention that resembles the example used by Glenn et al. (2016), according to which CO_2_ emissions are a cumulative effect of independent culturants and operants. The regulation of CO_2_ emissions through policymaking tends to address the scenario in which emissions are registered, and their effects appraised, cumulatively rather than independently from one another. Standing from the agendas of international regulators, this seems to be a shared approach and evidence is being summoned on the potential benefits of introducing behaviorally informed policymaking (OECD, 2017a, b, 2018). For example, BASIC (OECD, 2019) is a toolkit for applying behavioral insights to public policy problems. It represents an acronym that stands for behavior, analysis, strategies, intervention, and change. Some of the strategies that should juxtapose traditional policy interventions in behaviorally informed policymaking in the intervention phase include nudging, pushing, boosting, and curling, the last of which denotes “a paradigm of protection that attempts to weaken, remove and/or counter the psychological mechanisms identified by BI [behavioral insights] by trying to remove friction in choice architectures or counter illicit “nudges” by, for example, banning certain choice architectural features” (OECD, 2019, p. 118). For example, public policy could increase its effectiveness by removing behavioral costs and bureaucratic obstacles, providing timely information or prompts to action, and disclosing possible delayed consequences when selecting a specific course of action.

If policymakers can identify the contingencies of rational choice responsible for the maintenance of suboptimal consequences in a target reference group and place them into a cultural-selectionist perspective, interventions are more likely to last once System 1 (i.e., our intuitive approach) overtakes the analytical cognitive processing, and the designed controlling environmental contingencies fade into the background of our habitual choice architecture. Contextual considerations and analyses are critical for making meaningful contributions to social policy, and nudges can be useful to achieve the desired results if they can be readily implemented. For example, Fishbane et al. (2020) found that laypeople’s beliefs implied intentionality when they reduced failures to appear to court for low-level offenses through behavioral science: in particular, they achieved a 13%–21% reduction by enhancing salient information in the design of the form and sending text message reminders, which resulted in 30,000 fewer issued arrest warrants. In other words, salience is a form of stimulus control and acts through signaling and eliciting functions conditional on the agent’s attendance to the stimulus (da Rocha & Hunziker, 2020).

It is incredibly difficult to solve some of the contemporary (super-)wicked problems (e.g., saving the environment, eradicating mortal diseases, or globally defeating poverty) despite the efforts of the top behavioral scientists and using the best behavioral models that are available. Nevertheless, it seems that BA has not yet contributed enough to this mission, although the concepts of macrocontingency and macrobehavior were developed precisely to account for the complexity involved. For example, the work of Embry and Biglan reported by Wilson et al. (2014) consisted of implementing a community program aimed at reducing the sale of tobacco products to minors, which led to the formulation of a framework for intentional cultural evolution for both communities and organizations (Biglan & Embry, 2013). In a different policy area, behavioral systems analysis and organizational practices have informed the issues of climate change and global warming, signaling the contributions of behavioral science to contingency management for behaviors that involve the consumption of fossil fuels, motivating green choices concerning food preferences and diet, and addressing leaders’ organizational practices for enacting policies that facilitate a transition to sustainable practices (Alavosius & Houmanfar, 2020).

Goldiamond (1974/2002) developed the concept of *nonlinear behavior analysis* to reach beyond the descriptive linear logic of traditional functional analyses. It includes an analysis of different relationships between contingencies of alternative response patterns (de Fernandes & Dittrich, 2018); thus, the procedures that are necessary for an “understanding of why an alternative is preferred over another are: (a) to identify the set alternative contingencies that comprise the situation of choice; (b) to analyze the responses costs and the consequential benefits of all the alternative behavioral patterns” (p. 10). For example, the effects that nudging may have on an agent’s choice to get the COVID-19 vaccine depend not only on the relation of this choice behavior with its antecedent term, consequence, and response cost, but also with alternative behavioral patterns (i.e., not getting the vaccine, waiting to get the vaccine, or getting another vaccine brand or type included in the national vaccination program), provided that they do not have a strict preference for not getting the vaccine.

The outbreak of the COVID-19 pandemic may illustrate how a tiered model of intervention may function. Infection control started as advice for social interaction and soon turned into rules without sanctions, except for social contingencies for noncompliance. As the infection rate increased, rules started being enforced. Although society was locked down, enforced rules were followed by the delivery of consequences, including penalties. The dynamic shift between these levels tended to match the increases and decreases in infections. Thus, the effects on behavior with the least intrusive interventions may prevent more intrusive interventions in a long-term perspective.

Behavioral science should not limit its scope to the (ir)rationality of behavior: according to Ruggeri et al. (2019), “Behavioral sciences aim to draw justifiable, objective conclusions about these inconsistent behaviors through systematic study” (p. 60; emphasis in original). Behavioral science, consistently and in concert with BA, should address all forms of behavior understanding and modification. By encompassing conceptual, experimental, and applied settings, behavioral science may increase its scope from the level of communities and organizations to public policymaking. For example, nudging represents a tactic of common interest that may unite different approaches and advance the field as one. Several scholars adhering to the BA approach have taken nudging as a standpoint to inquire further about the relation between BE and BA and have outlined promising avenues for further cooperation (e.g., da Rocha & Hunziker, 2020; Furrebøe & Sandaker, 2017; Rachlin, 2015; Tagliabue et al., 2017), including relational frame theory (Tagliabue et al., 2021).

As the consequences of one agent’s choices affect the choices of other agents, the effectiveness of (social) nudging interventions can be regarded as the product of cumulative and interlocked choices at the policymaking level, which warrants the analysis of macro- and metacontingencies. If the choice environment is not explicitly designed, whether an agent’s choice behavior is rational or not may be maintained after its first spontaneous or elicited occurrence by the traits of the cultural setting. Hence, rational choice may be regarded as instruction-governed behavior or as environmentally or culturally shaped, regardless of the instruction. For example, federal interventions for reducing greenhouse gas emissions and fighting climate change (e.g., Bonner et al., 2021) and the educational and social outcome framework termed school-wide positive behavioral interventions and supports (PBIS; e.g., Horner & Sugai, 2015) may characterize two timely and relevant products of both cumulative and interlocked choice.

Embracing a systems perspective extends the outreach of behavioral interventions regardless of whether they aim to provide one-time-use strategies for correcting the course of action (i.e., nudging) or educate agents towards more sensible outcomes (i.e., boosting). In terms of return on investment, the costs and savings of behaviorally informed policymaking outweighs traditional policymaking (i.e., largely based on the use of economic incentives and disincentives); however, the former does not comprise a universal replacement for the latter and may rather comprise an efficient alternative (Arno & Thomas, 2016). For example, educational campaigns for influenza vaccination uptake and the provision of incentives and training for energy conservation proved to have relatively high effectiveness, second only to the most effective nudge: planning-prompt and social norms, respectively (Benartzi et al., 2017).

### Ethics of Behavioral Influence and Control

Empowering researchers and policymakers with tools of behavioral control, even of the softest kind, bears ethical considerations that deserve closer analysis. Among the recipients of behavior change policies, it has been argued that citizens’ favorable views of different degrees of paternalistic policies from their regulators vary as much in Europe (Reisch & Sunstein, 2016) as they do worldwide (Sunstein et al., 2017; Sunstein et al., 2018). Some of the issues concern the justification for disclosing or concealing information on the methods of nudging (Marchiori et al., 2015), and the legitimacy of whether the proposers or the recipients of nudges should have the right to judge their applicability for forming healthier habits (Sugden, 2017).

However unobtrusive, nudging represents a tactic of behavioral influence and several scholars have evaluated and disclosed the ethical considerations of nudging concerning the behavior-altering reproach towards agents’ preferences (Selinger & Whyte, 2011; Sugden, 2017; Sunstein, 2015a, 2018; Thaler & Sunstein, 2003). Other ethical considerations have touched on the political debate concerning the justification of whether nudging interventions should be embedded in public policymaking at all (Frantz, 2018; Sunstein, 2015b, 2016a; Sunstein et al., 2017). The OECD (2017b) reported that ethical barrierswere not perceived to hinder the enactment of behavioral insights in public policy; on the other hand, ethical concerns were integrated into policy design and implementation, conforming to public interests and the right of agents to self-determination.

Like other cases of rule-governance, such as compliance to the penal code and the obligation of paying taxes, levels of behavioral interventions may be ranked from least to most invasive depending on the level of coercion involved (Sidman, 1989). A four-tiered division may help and should include: (1) the absence of any influence on the agent (i.e., no guidance, not “neutral”); (2) social nudges that make use of other people and reference points surrounding the agent (e.g., commercial campaigns, norms, implementation of intentions (Costa & Kahn, 2013); (3) agent-based nudges that exploit the moment of choice, such as default options, the use of contextual cues, their timing, warnings, and the manipulation of convenience and ease costs (Sunstein, 2014a); and (4) the introduction of rules, which include different levels of enforcement and whose consequences increase in severity if proven ineffective (Ostrom, 2000).

As suggested by historical evidence, in general, BA is not seen as a driving force in the broader behavioral science approach. For example, Fawcett et al. (1988) underlined the strength of behavior analysts’ functional models at the service of public policymaking and influence, while also stressing the roles of task preparation and cross-sector collaboration. However, BA is a clear case of scientific insularity because the common object of inquiry is the understanding, prediction, and influence of behavior. Although BA is not synonymous with behavioral science, although the two may have erroneously been used interchangeably at times, it can hardly be argued that BA is not a constituent natural science of behavioral science. It is interesting that Rachlin (1989) compared the terminology used in operant-choice experiments and economics and found that in several instances it was equivalent: for example, reinforcers correspond to goods commodities, punishers to “bads” commodities, and, when schedules of reinforcement are in effect, matching corresponds to maximizing under constraint. Further points of contact and cooperation between these fields should develop the common premises of the effects of the environment on choice behavior. Historically, BA and BE have addressed choice behavior relatively independently of each other, or as Frid-Nielsen and Jensen (2021) reported in their reference analysis, the role of several behavioral psychologists who contributed to the historical foundations and development of BE (e.g., Skinner, Herrnstein, Hursh) was overlooked. Behavioral insights are a new and exciting field in which more conceptual advancements and empirical studies from behavioral science are called for. Nevertheless, they have developed and spread mainly independently from the field of BA.

In BE, it is commonly understood that neither nudges nor BE boosts should interfere with one’s agency and free will, which are concepts that are generally rejected according to the view of radical behaviorism. No incentive or punishment, economic or in terms of time, effort, trouble, or social sanction is directly imposed (Sunstein, 2014a, 2014b). However, *directly imposed* appears to miss some of the dynamics of choice in the behavior analytic literature. As Skinner (1971) put it, “Freedom is a matter of contingencies of reinforcement, not of the feelings the contingencies generate” (p. 42). The manipulation of contingencies has no positive nor negative value per se: insofar as contingencies influence behavior, both learning history and environmental design may affect the agent’s perception of freedom.

In *Beyond Freedom and Dignity*, whose 50^th^ anniversary since its publication happens to occur at the time of writing, Skinner (1971) included free will and the absence of control as conditions of freedom: the latter was operationalized as the absence of aversive control of behavior, which may question the degree of freedom that nudging and BE boosting involve as behavior changes as a function of choice between contingencies. The concept of freedom in Skinner has been linked to self-knowledge, self-control, and countercontrol (Dittrich, 2010). Thus, BE boosting retains more freedom than nudging because of a higher degree of disclosed self-knowledge in current and future occurrences. BA boosting retains even more freedom compared to the other approaches with respect to the provision of positive reinforcement contingent on, for example, displaying self-control and maintaining the policy initiative. However, nudging and both forms of boosting are viewed similarly insofar as the agent’s behavior may punish policymakers’ initiatives or set them on extinction. For example, nudge policies may have unintended adverse effects, choosers may have strong contrary antecedent preferences, or agents may develop counternudging measures; all of which will likely lead to rejecting the policy intervention (see Sunstein, 2017).

According to Staddon (2018) an agent’s repertoire is limited by its history, which in turn represents the boundaries of variability to which a rational agent has been exposed; in his view, nudges are an example of applying frames for social engineering purposes (da Rocha & Hunziker, 2020). This view can be operationalized into degrees of freedom and coercion developed several years earlier by Goldiamond (1965), who defined freedom in terms of the number of available response alternatives. Nudges can be perceived as more or less “controlling” (Thaler, 2018), but nudges and other behavioral interventions may exert insufficient control over agents’ choices. Although historically and methodologically different, nudging and boosting are functionally similar insofar as they can alter the value of the consequences of choice. Based on the idea proposed by Sunstein (2016a, 2016b) that autonomy is not an absolute concept, nudges “would help to make people at least relatively autonomous [emphasis in original]” (da Rocha & Hunziker, 2020, p. 144).

Baum (2017) defined freedom as the condition of having choices and not being punished for making them, which is consistent with the definition of nudging and both forms of boosting. Furthermore, Catania (1980) arranged different competing schedules of reinforcement and recorded their effects on experimental subjects’ possibilities and values of freedom of choice. Although partially agreeing to their standpoints on freedom, Goldiamond (1976) added the analysis of genuine choices and critical consequences that a society or an institution may have on their members to the appraisal of coercion. Critical consequences have a powerful behavioral control effect when added or removed and they should be available for different patterns of behavior beyond alternative contingencies (de Fernandes & Dittrich, 2018). Formally, programming critical consequences seems in antithesis with the definition of nudging and other behavioral interventions that alter antecedent terms: choice is retained without affecting the economic incentive system, which does not warrant a coercion-free choice architecture.

The concepts of institutionally instigated coercion and institutionally opportune coercion comprise behavior-analytic accounts of power relations between controlling policymakers and controlled agents and are particularly valuable for discussing the relationship between coercion and institutions. According to de Fernandes and Dittrich (2018), institutionally opportune coercion “provides the possibility of establishing socially desirable or acceptable practices, but also creates opportunities for establishing abusive practices” (p. 15), whereas the libertarian-paternalistic foundations of nudging and the educative aim of BE boosting lack the ability to make consequences critical while arranging the set of contingencies that grant them (see Goldiamond, 1976). As policymakers embed nudges and boosts in their programs, they stipulate the behaviors required by the agents for gaining access to their designed consequences. However, they do so without establishing the conditions that make the consequence critical: for example, the contingencies for having access to education, achieving herd immunity, or experiencing the catastrophic effects of global warming.

There seem to be ethical limits to the approximation of nudging (and boosting) and BA. The former rests on the libertarian approach to freedom of choice and personal responsibility, whereas the latter takes a deterministic stance and denies their possibility according to radical behaviorism. Moreover, the liberal take of Rakos (2004) and the more libertarian perspective of Staddon (2003) lie somewhere between those extremes. Nudging provides valuable and relevant means of behavior change; it is a promising way forward if the choices that are prompted can be sustained and applied to novel settings. For example, nudges may provide guidance in situations where a choice needs not to be weighed thoroughly, or beyond one-time decisions, such as planning retirement savings or signing up to the national organ donor registry. The next frontier of nudging may feature the withdrawal of the nudge serving as the antecedent term in the contingency and the delivery of reinforcement if the nudge policy is followed, instead, which is consistent with BA boosting. Conversely, policymakers should prefer boosting to nudging when agents’ goals are uncertain, heterogeneous, or conflicting, if governments do not act benevolently or fail to safeguard agents from the private sector’s economic interests, and to foster generalizable and lasting behaviors (Hertwig, 2017).

Similar to studies of delay discounting, forthcoming experimental efforts should address the relation between magnitude and delay of rewards. Although nudges represent “means of bringing behavior under the control of wide and abstract reinforcer contingencies” (Rachlin, 2015, p. 198), further studies may investigate the precision and sensibility of nudging alternatives and include the analysis of macro- and metacontingencies into diagnostic models of policymaking (e.g., MINDSPACE; Behavioural Insights Team, 2010; *Define, Diagnose, Design, Test*; Ideas42, 2017). For example, Couto et al. (2020) identified six steps for developing a system response to the COVID-19 pandemic informed by a metacontingency analysis: (1) define a common behavioral goal; (2) shift from defective to complete protective macrocontingencies; (3) map roles and identify agents to complete any contingency gaps; (4) connect and coordinate interlocking behavioral contingencies; (5) highlight the aggregate product; and (6) favor cultural consequences. Public policymakers can draw from these steps and implement them in any other domain where problems require coordinated action and their possible solutions feature a high degree of complexity (e.g., climate, health, education).

Cognitive biases are systematic deviations from instances of rational choice: their meaning and role are entwined with some definitions of nudging, although they formally preceded the debate of whether biases should be considered motives for nudging. The same may be stated about heuristics, which represent cognitive shortcuts that work well most of the time, or else they would not have survived throughout both our phylogenesis and ontogenesis. Although they have not been formally addressed in this study, heuristics are the product of the selection process of an agent’s learning history or, by extension, of his or her reference group. Whether a choice is perceived as right or wrong, or whether the feedback of a certain course of action is not transparent, erroneous heuristics and biases may prevail and be transmitted. However, according to a post-Skinnerian account of language and cognition, a bias represents “a product of our processes of symbolic derivations that are contextually controlled and that occasion verbally controlled behavior” (Tagliabue et al., 2021, p. 15). Thus, public policies should transform biases and other displays of supposedly irrational behavior to serve new and more sensible behavioral and cultural repertoires, rather than dismissing them as unwanted noise in their analyses and solutions.

Another avenue of further research and collaboration includes behavioral decision research, which has greatly influenced the making of BE has been further advanced thanks to the innovative methods and findings typical of decision neuroscience. The relatively new field of neuroeconomics is the result of this synthesis, establishing a neuroscientific account of the interplay between cognition and affect, and automatic and controlled processes of choice (Camerer et al., 2005). It may be argued that these processes can account for why boosts (or nudges altogether) might not be sufficiently powerful tools of cognitive or behavioral control. Self-nudging (Reijula & Hertwig, 2020) comprises a spinoff of the BE tradition concerned with the creation and implementation of self-rules to overcome self-control problems that can be scaled up to public policy through functional and methodological contributions from BA. Moreover, self-nudges are self-imposed and virtually immune from ethical critiques of behavioral control and influence imposed by an external policymaker, regulator, or other authority. Hence, verbal behavior has a primary mediating function in the acquisition and transmission of behavior and cultural practices. Without neglecting the other two levels of selection illustrated by Skinner (1981; viz., genetic selection and operant conditioning), a selectionist perspective of the sociocultural units of analysis seems most apt.

Whereas macrocontingencies are limited to collective responses that may incidentally put groups in contact with the sum of individual consequences, metacontingencies “focus on the selection of coordinated responses that enable groups to achieve shared goals” (Couto, 2019, p. 8); this is where BA applied to public policy can contribute the most. In turn, the concept of rationality and the findings from nudging and boosting research provide practical and cost-effective means of intervening on operants and culturants. The claim that “[w]hen feedback does not work, we may benefit from a nudge” (Thaler & Sunstein, 2008, p. 75) seems to find ample support in the tradition of BE. However, principles of reinforcement posit that feedback may suffice and rational agents’ choice behavior need not be nudged or boosted in line with the potentially reinforcing consequences programmed by policymakers.

## Footnotes

1 The colloquial term has longer roots because the origin of nudge may be dated 1665–1675 as a variant of dial. *(k)nidge*, akin to Old English *cnucian*, *cnocian*, to knock (source: https://www.dictionary.com/browse/knock).
